# Centralization and transport of critically ill pediatric patients

**DOI:** 10.3389/fped.2025.1601875

**Published:** 2025-06-04

**Authors:** Ryo Kamidani, Hideshi Okada

**Affiliations:** ^1^ Department of Pediatric Critical Care, Saitama Children’s Medical Center, Saitama, Japan; ^2^ Department of Emergency and Disaster Medicine, Gifu University Graduate School of Medicine, Gifu, Japan; ^3^ Center for One Medicine Innovative Translational Research, Gifu University Institute for Advanced Study, Gifu, Japan

**Keywords:** pediatric intensive care units, specialized pediatric transport teams, transport, centralization, helicopter emergency medical services, telemedicine, patient stabilization, pediatric patients

## Abstract

**Background:**

Caring for critically ill pediatric patients requires specialized expertise, centralized facilities, and efficient transport systems. The centralization of pediatric intensive care units in tertiary centers has enhanced clinical outcomes, resource efficiency, and standardized care. In this study, we provided an updated review of the increase in need for specialized pediatric transport teams.

**Methods:**

We searched PubMed for peer-reviewed literature on the treatment and transport of critically ill pediatric patients, as well as websites of government agencies involved in reporting population prospects. The following search terms were used: pediatric intensive care units, specialized pediatric transport teams, centralization, and helicopter emergency medical services. Thereafter, an inductive qualitative content analysis was performed.

**Results:**

High-volume pediatric intensive care units are associated with lower risk-adjusted mortality rates and more efficient resource utilization. However, over-centralization may reduce quality. Effective patient transport depends on skilled personnel, coordination, and stabilization, regardless of the team's composition. Therefore, transport methods should be selected based on a patient's condition, distance, and regional resources. Although helicopters enable rapid transport, they pose risks such as patient-related adverse events, operational hazards, and high costs. Additionally, recent studies questioned the “golden hour” concept, emphasizing stabilization and timely care over speed. Telemedicine plays a crucial role in reducing unnecessary transfers, optimizing resources, and improving access to specialized care.

**Conclusions:**

As aging populations and declining birth rates reshape healthcare needs, the demand for specialized pediatric transport and telemedicine increases. Future strategies must address regional disparities, enhance cost-effectiveness, and integrate advanced technologies such as artificial intelligence to ensure equitable and high-quality pediatric care.

## 1 Introduction

Caring for critically ill patients requires specialized intensive care knowledge and skills, as well as considerable human and material resources, including a medical workforce capable of handling shifts, specialized medical equipment, and facilities. A United States cohort study reported an incidence rate of 9.3 admissions per 1,000 person-years for children in the first adult intensive care unit (ICU) (1). Given the low incidence of critically ill pediatric patients, their care should ideally be managed in a pediatric intensive care unit (PICU), staffed with specialists in pediatric emergency and intensive care (2). In most countries, pediatric intensive care is centralized in tertiary centers (3), and larger, high-volume centers with more annual admissions have been reported to achieve better clinical outcomes (4).

**Figure 1:**
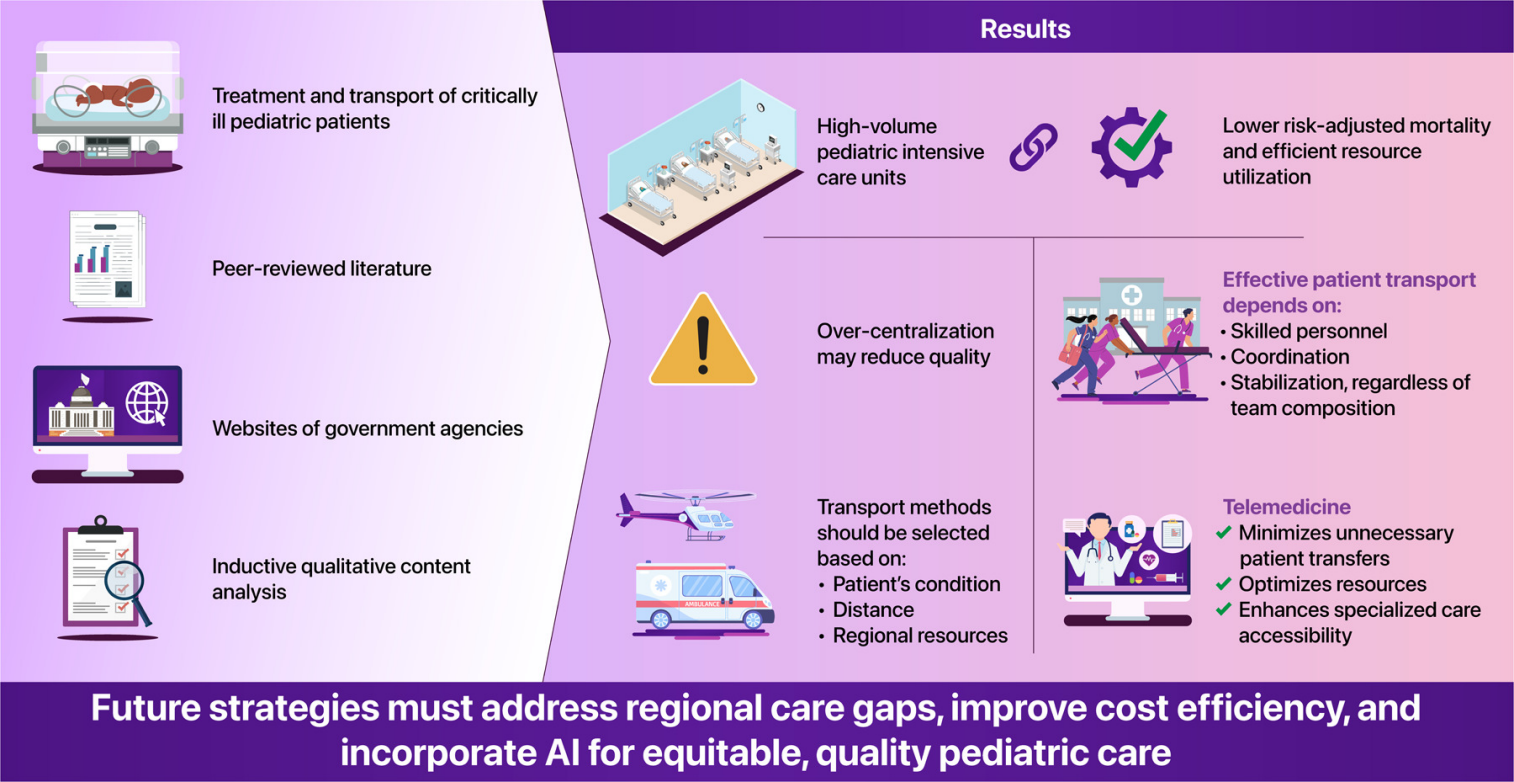
Conceptual framework of the centralization of pediatric intensive care. The figure highlights the key components and interactions involved in the regionalization and transport of critically ill pediatric patients. Centralization into tertiary PICUs, supported by specialized transport teams and telemedicine, is associated with improved outcomes and efficient resource utilization.

To effectively centralize care for critically ill pediatric patients at tertiary centers, regional systems typically integrate pre-hospital emergency medical services (EMS) transport protocols with adult-oriented emergency networks while simultaneously assessing the entire community's medical resources. Certain regions also have pediatric specialist transport teams from tertiary centers for interhospital transfers. Furthermore, comprehensive planning is necessary, including wide-area transport using helicopters and fixed-wing aircraft carrying physicians.

This review discusses the transport needs of critically ill pediatric patients, the methods and strategies to meet these needs, and the future of pediatric intensive care and specialized pediatric transport medicine. Although certain sections refer to neonatal transport from a transport medicine perspective, it should be emphasized that the present review is confined to the transport of pediatric patients.

## 2 Expansion of PICUs and benefits of centralization

Everyone has the right to receive adequate quality care when seriously ill or injured. In the 1980s, as part of the expansion of medical services for pediatric patients, PICUs expanded rapidly, primarily in developed countries (5). Centralizing critically ill pediatric patients in PICUs offers multiple benefits, including improved clinical outcomes, enhanced working conditions for healthcare providers through optimized resource use, standardization of clinical practices, facilitation of large-scale clinical research, and more efficient training of pediatric intensive care specialists. (Figure 1 illustrates the conceptual framework and key components involved in the centralization of pediatric intensive care.)

Larger PICU scales (number of patients and beds) are associated with lower risk-adjusted mortality rates (6, 7). However, several reports indicate no relationship between the number of PICU beds and the length of hospital stay (LOS). When comparing Victoria, Australia (where centralization has been progressing for some time), with Trent, England, the risk odds ratio for death among critically ill children in the English county of Trent was reported to be 2.09 (1.37–3.19). In contrast, LOS in PICUs was shorter in Victoria (2, 6, 8). Additionally, the presence of intensive care physicians, residents, and collaborative care has been reported as factors relating to shorter LOS (2). Centralizing patients into large-scale PICUs offers numerous benefits, making it desirable to establish a specialized transport team for active patient transport between hospitals. Optimizing the allocation of PICU resources requires a nuanced approach that considers demographic trends, the ratio of PICU beds to total hospital capacity, and the regional adequacy of the critical care infrastructure. Strategic planning in these areas is critical to ensure equitable access to high-quality pediatric intensive care.

What are the differences in treatment outcomes between PICU and adult ICU patients? When comparing a single condition, such as severe sepsis, the mortality rate among patients admitted to the PICU in the United States is 14.4%. In contrast, the rate of those admitted to the adult ICU ranges from 14.9% to 34.3%, indicating higher mortality in the ICU (9, 10). Zakutansky et al. conducted a database study of young adults aged 18–26 years with severe sepsis in the United States and reported the treatment outcomes of patients treated in PICUs or medical/surgical ICUs. They considered this study population unique because young adults were eligible for treatment in both PICUs and ICUs. Patients treated in PICUs were found to have a higher incidence of comorbid conditions such as genetic disorders, hematologic disorders, malignant tumors, and neuromuscular disorders, resulting in higher in-hospital mortality rates (11).

## 3 Evolution and optimization of pediatric transport systems

Patient transport medicine, often developed as part of military medicine during wartime, has been characterized by organized operational systems. The transport of neonatal patients was developed in the late 1960s and early 1970s, preceding the transport of critically ill pediatric patients (12, 13). During this period, neonatal intensive care focused on centralization and regionalization, which were reported to improve mortality rates in critically ill neonatal patients (14).

However, since no similar improvements in morbidity, such as neurodevelopmental disorders, have been observed, the establishment of fetal diagnosis has led to active maternal transport to tertiary centers, which has increasingly facilitated perinatal centralization (15). Thus, from the perspectives of centralization and regionalization, modern pediatric critical care transport can be traced back to the foundational practices in neonatal medicine.

As the centralization of pediatric intensive care progresses, the demand for pediatric critical care transport teams increases. Previous studies have shown that transferring children by specialist teams improves outcomes and reduces unexpected events (such as airway events, cardiac arrest, sustained hypotension, equipment failure, and deterioration of patient status) compared to basic EMS, or medical staff teams making referrals (16–18).

During interfacility transport, destabilizing factors, such as vibration (acceleration and deceleration forces), noise, environmental temperature fluctuations, and confined spaces, pose significant risks for severe medical incidents. However, no globally standardized, evidence-based guidelines exist for the equipment and management of pediatric patients during transport. A cohort study in the UK and Ireland reported that approximately half of pediatric patients requiring interfacility transport were infants, with respiratory diseases accounting for most cases (19). Similarly, Crow et al. reported that the highest rate of PICU admissions occurs within the first year of life, with a subsequent decline, and that respiratory diseases remain the most common reason for this admission across all age groups (1). Therefore, ensuring quality emergency care and airway management for infants is critical for the safe interfacility transport of pediatric patients. Pediatric critical care transport team members must possess advanced skills in assessing and managing these patients effectively.

The role of physicians in specialized transport teams remains debated. Shinozaki RM et al. reported that including a physician during interfacility transport of critically ill pediatric patients increased response time (defined as the time from the initial call to the PICU, to arrival at the referring hospital) by a median of 0.26 h (interquartile range: 1.4–2.9 h). However, this inclusion had no significant effect on mortality or LOS in the PICU (20). Similarly, Belway et al. reported no correlation between transport time and mortality in adult patients with cardiac diseases. However, longer response time was associated with shorter hospital stays (21). Furthermore, a cohort study in the UK and Ireland revealed that interfacility transport led by a senior physician (consultant) or involving other ICUs was significantly associated with PICU mortality (19). These findings suggest that physician presence during transport does not necessarily guarantee favorable outcomes. However, this interpretation warrants caution. Ramnarayan et al. acknowledged that the observed association between consultant-led transports and mortality cannot be fully explained, indicating the possible presence of residual confounding factors (19). This may be attributed to the fact that, in many clinical scenarios where a physician, particularly a senior physician, is present during transport, the patients are often more severely ill. At the very least, these results should not be interpreted to imply that less experienced physicians are preferable. While nurses or respiratory therapists with advanced skills effectively manage interfacility transport, further research is needed to better understand the optimal composition of transport teams (22). Moreover, fostering precise communication between hospitals and utilizing response time to stabilize a patient's overall condition through the efforts of staff at the referring hospital may improve patient outcomes (23).

## 4 Selection and optimization of pediatric transport modalities

Patient transportation means include ambulances, helicopters, fixed-wing aircraft, trains, and ships. While emergency medicine physicians and intensivists often ensure their ability to use them in various situations, the selection is determined by the physician at the transfer hospital or emergency department. The crucial aspect in selecting the mode of transportation, whether for transport from the scene or interhospital transfer, is to minimize out-of-hospital time and connect the patient to definitive treatment as quickly as possible. Recent studies have challenged the “golden hour” concept in pediatric and neonatal patients’ emergency transport. They emphasize the importance of appropriate treatment and stabilization before transfer, rather than transport speed alone (24). In interfacility transport, delays in receiving definitive treatment at tertiary care centers often stem from prolonged stays at the referring hospital. Therefore, beyond individual efforts, establishing well-organized interfacility transport systems is essential (25).

Ambulances owned by hospitals, or exclusively used by physicians (referred to as “Dr. Car” in Japan), are the most common patient transport mode operated by EMS. Their advantages include high mobility, the ability to swiftly reach any location, even in adverse weather conditions or at night, and the fact that they are the fastest mode of transport for short distances (up to approximately 20 km). Another advantage is that, since the vehicle operates on roads, it is possible to temporarily stop the vehicle to avoid vibrations during procedures that require stability. However, treatment inside an ambulance may be limited by shaking and vibrations caused by road conditions, and delays can arise owing to traffic congestion, speed limits, or geographical barriers (26).

Various authorities and operational methods govern emergency helicopters, including fire departments, police, and helicopter EMS (HEMS). The primary advantage of HEMS is its ability to quickly deploy specialized physicians (emergency physicians) to the scene. In certain regions and countries, pre-hospital emergency services are staffed by paramedics and nurses. Although regional variations exist, helicopter transport is generally the fastest mode of patient transfer over medium distances (approximately 50–200 km) (27). However, operations are significantly impacted by weather and wind conditions. For example, in Japan, helicopters must operate under visual flight rules, prohibiting nighttime operations. Additionally, the complex processes of mission approval and preflight checks can delay transportation (28). In contrast, fixed-wing aircraft can cover longer distances than helicopters and are suitable for long-range transport across prefectures. They are less affected by weather conditions and can be pressurized during flight. However, they require a runway for takeoff and landing, and transportation from the airport to the medical facility must be arranged in advance (29).

HEMS have been shown to offer shorter transport times than ground transport, except for short distances (approximately 20 miles) (27, 30, 31). Although shorter transport times may seem advantageous, a study reported that only 43% of critically injured pediatric patients experiencing trauma required time-sensitive interventions within 4 h of arrival at a tertiary care center (32). Another study indicated that 35% of pediatric patients experiencing trauma needed intervention within 6 h of transfer to a pediatric trauma center. However, no significant outcome difference was observed between ground and air transport (30). Although the benefits of specialized transport teams are clear, these findings suggest the potential overuse of HEMS. Typically, HEMS should be reserved for patients requiring urgent, time-sensitive interventions or for those for whom ground transport is not feasible owing to specific circumstances.

## 5 Risks and cost-effectiveness of HEMS

The HEMS is an indispensable service for the centralization of medical resources. However, it carries three primary risks: (1) patient complications during transport, (2) operational safety risks for patients and team members, and (3) financial risks associated with its operation.

A study conducted in Thailand on pediatric patient transport reported higher rates of complications, such as hypotension and cardiac arrest, in HEMS compared to ground transport. The authors suggested that this finding may be influenced by HEMS being chosen for more severely ill patients and the study's inadequate adjustment for illness severity (31). From an operational safety perspective, the crash rate for HEMS is approximately 6,000 times higher than that for civilian aviation, making it one of the most hazardous occupations in the United States, second only to commercial fishing. Additionally, the primary causes of HEMS accidents include weather conditions, darkness, inadequate landing zones, terrain, and mechanical failures. Nearly half of HEMS crashes occur at night, probably owing to poor visibility and pilot fatigue during night shifts (33). In mountainous areas, rapidly changing weather, clouds, fog, and thunderstorms further complicate flight operations. In Japan, where HEMS began full-scale operations in 2001, regulations restrict flights to daylight and clear weather owing to visual flight requirements. Consequently, Japan has not yet experienced fatal HEMS crashes.

From a financial perspective, no studies have specifically focused on the cost-effectiveness of HEMS in pediatric transport. Delgado et al. suggested that for HEMS to maximize cost-effectiveness, mortality must be reduced by at least 15%, or long-term disability outcomes must be quantitatively improved (34). Increasing the number of transfers, including those with less severe conditions, and promoting centralization may be the keys to improving cost-effectiveness. Owing to their economic burden, HEMS operations are often subsidized by regional or national government funding. Enhancing transparency regarding cost-effectiveness and implementing cost-reduction strategies are essential for advancing pediatric transport medicine.

## 6 Challenges of centralization and future directions

Despite the benefits of centralizing pediatric patients and their means of transportation, several studies have highlighted certain challenges. Marcin et al., using data from 15 PICUs in the United States, performed an instrumental variable analysis employing mixed-effects and hierarchical modeling (4). They reported that for every additional 100 annual PICU admissions, the severity-adjusted odds of mortality decreased by 0.68 (95% confidence interval: 0.52–0.89). Bed capacity and staffing levels were not examined; however, units with higher yearly admission volumes demonstrated superior clinical outcomes. The lowest mortality was observed in PICUs admitting approximately 1,500 patients per year. Conversely, centers admitting well above 1,500 patients annually showed a modest increase in mortality, warranting caution in interpreting very-high-volume performance. Large PICUs have higher bed utilization efficiency than smaller ones due to fewer irregular fluctuations in bed demand. However, if the number of inpatients yearly is too high, maintaining the quality of care may become impossible. The presence of intensive care physicians and residents, as well as collaboration in care, has been reported as factors related to shorter LOS (2).

A secondary analysis of the global SPROUT study on severe pediatric sepsis reported that PICUs in Europe have fewer beds than those in the United States (11 vs. 24 beds, *p* < 0.001) (35). Furthermore, European PICUs admit more critically ill children at a younger age, and a higher proportion of admissions come from general wards. Each country has unique geographic circumstances. In countries with large land areas, regions with low population densities face challenges in accessing small regional hospitals, let alone hospitals with large-scale PICUs. Conversely, countries with small land areas, such as Japan, have many small- and medium-sized general hospitals, making effective centralization difficult. In some cases, pediatric medical care exceeds capacity.

The global population reached five billion in 1986, six billion in 1998, seven billion in 2010, and eight billion in 2022. However, the annual growth rate has steadily declined since peaking in the late 1960s (36). According to the United Nations World Population Prospects, the global population is expected to grow over the next 50–60 years, reaching approximately 10.3 billion by the mid-2080s before gradually declining during the remainder of the 21st century. Currently, more than half of the world's countries have a total fertility rate below the population replacement level of 2.1, with population growth in parts of Asia and Africa heavily influenced by demographic changes. By the mid-2030s, the global population aged 80 years and older is projected to reach 265 million, surpassing the number of infants under 1 year of age (37).

While the global population growth is slowing, Japan is experiencing the most pronounced decline in birth rates and the most rapid population aging. The Olympic Games served as a symbolic turning point in their social change. In 1964, when the Tokyo Olympics were first held during the period of high economic growth, the number of births was approximately 1.71 million, and the total fertility rate was 2.05. Conversely, in 2021, the year of the second Tokyo Olympics, Japan had become a mature economy and super-aging society, with the number of births plummeting to approximately 810,000, and the total fertility rate dropping to 1.30 (further declining to 1.20 in 2023). This represents a 53% decrease in births over 57 years. Although the country has long benefited from the development of modern medicine, good public health, widespread use of maternity health records and a neonatal mortality rate (one of the lowest in the world at 0.8 per 1,000 births), it is facing a low birthrate and an aging society at an unprecedented speed in the world (38). Tragically, Japan is approximately 100 years ahead of the global average in population aging.

The decline in the labor force and shrinking domestic markets will likely result in slower economic growth, consolidation of healthcare institutions, and reduced social security programs. Furthermore, as depopulation progresses, certain rural areas may lose their ability to maintain sufficient infrastructure. Japan has historically had one of the lowest numbers of physicians per capita among the countries of the Organization for Economic Cooperation and Development. However, efforts to increase medical school admissions since the 2000s have brought their physician-to-population ratio closer to that of Canada and the USA (39). According to the Ministry of Health, Labour and Welfare, the number of board-certified pediatricians increased from 13,145 in 2000 (8.9 per 100,000 population) to 17,781 in 2020 (14.1 per 100,000), reflecting the overall physician growth (40). Nevertheless, as the pediatric population continues to contract, projections show that the pediatric workforce will peak within the next decade and decline thereafter. During this period of population redistribution and municipal mergers, pediatric emergency services are likely to become more centralized, leading to an increase in the number and distance of specialized pediatric transport. Consequently, the demand for specialized pediatric transport teams is expected to remain high in the foreseeable future.

Telemedicine has recently advanced beyond education and research to include remote consultations and care. Chronic conditions such as asthma, diabetes, and epilepsy are particularly well-suited for telemedicine (41, 42). In the critical care domain, PICU-based telemedicine programs offering live interactive consultations with rural adult ICUs have been reported to provide high-quality care with high levels of satisfaction (43, 44). Telemedicine for pediatric patients admitted to general pediatric wards or adult ICUs reduces unnecessary patient transfers, optimizes healthcare resources, and alleviates the burden on families (45).

Many intensive care guidelines set lower age limits for admission to adult ICUs. Eighteen years is generally considered the standard threshold; however, some recommendations suggest that critically ill patients as young as 16 years should preferably be managed in PICUs, whenever feasible (46, 47). Recent literature proposes that admission to adult ICUs may be appropriate for patients aged 12 years or older, provided pediatric medical devices are available (48). However, telemedicine programs may lower the minimum age limit for ICU admission. Although the studies linking PICUs and adult ICUs in rural areas did not specify clear age criteria, they included infants. Further research is needed to determine the acceptable age range that ensures safety, as conditions vary by country and facility (43, 44).

Admission of pediatric patients to adult ICUs requires the availability of appropriate equipment and supplies, including blood pressure cuffs suitable for pediatric body sizes, small-bore endotracheal tubes, pediatric ventilator circuits, infusion pumps capable of delivering low syringe volumes, and dialyzers with reduced membrane surface areas. In facilities lacking such resources, patient safety cannot be ensured even with telemedicine support. Therefore, infants and young children should be transferred to PICUs or to facilities equipped with the necessary pediatric infrastructure whenever possible. Advancements in telemedicine equipment, such as video conferencing units and connected medical devices (e.g., examination cameras, stethoscopes, monitors, and ultrasound machines), along with the integration of artificial intelligence technologies, are expected to drive further improvements in quality and cost efficiency for healthcare. As these technologies evolve, global adoption of telemedicine is expected to expand significantly.

Overall, centralization of pediatric intensive care has been shown to improve outcomes, resource efficiency, and care standardization. However, regional differences and logistical challenges require tailored strategies. Specialized pediatric transport teams are critical for safe, effective transfer, with success depending on skilled personnel and well-organized systems rather than transport speed alone.

Advancements in telemedicine and artificial intelligence, along with continued investment in infrastructure and workforce development, will be key to addressing the growing demand for specialized pediatric transport, particularly in an aging and declining population. Achieving balanced cost-effectiveness, safety, and accessibility will be essential for sustaining high-quality care.
